# Dimerization of Peptides by Calcium Ions: Investigation of a Calcium-Binding Motif

**DOI:** 10.1155/2014/153712

**Published:** 2014-09-14

**Authors:** Azadeh Jamalian, Evert-Jan Sneekes, Lennard J. M. Dekker, Mario Ursem, Theo M. Luider, Peter C. Burgers

**Affiliations:** ^1^Department of Neurology, Laboratory of Neuro-Oncology, Erasmus Medical Center, 3015 GE Rotterdam, The Netherlands; ^2^Thermo Fisher Scientific, 1046 AA Amsterdam, The Netherlands

## Abstract

We investigated calcium-binding motifs of peptides and their recognition of active functionalities for coordination. This investigation generates the fundamentals to design carrier material for calcium-bound peptide-peptide interactions. Interactions of different peptides with active calcium domains were investigated. Evaluation of selectivity was performed by electrospray ionization mass spectrometry by infusing solutions containing two different peptides (P_1_ and P_2_) in the presence of calcium ions. In addition to signals for monomer species, intense dimer signals are observed for the heterodimer ions (P_1_ ⋯ Ca^2+^ ⋯ P_2_) (⋯ represents the noncovalent binding of calcium with the peptide) in the positive ion mode and for ions ([P_1_-2H]^2−^ ⋯ Ca^2+^ ⋯ [P_2_-2H]^2−^) in the negative ion mode. Monitoring of the dissociation from these mass selected dimer ions via the kinetic method provides information on the calcium affinity order of different peptide sequences.

## 1. Introduction

Calcium is one of the most abundant cations in living organisms [1, 2]. As an intracellular signaling ion, Ca^2+^ plays crucial roles in an array of cellular functions from fertilization, muscle contraction, and cell differentiation/proliferation to apoptosis and, in the case of dysregulation, cancer and neural diseases [3–6]. The impact of monitoring calcium in proteins can be extremely high. For example, mutations in calcium ion transport proteins can disrupt channel functions and have been associated with various diseases, like Alzheimer's disease [6]. However, Ca^2+^ does not act alone. Many cells contain a variety of cytosolic calcium-binding proteins (CaBPs) which either modulate or mediate the actions of this ion [7–9]. Depending on the role and cellular locations of the CaBPs, their affinities may vary by as much as 10^6^-fold [10]. These proteins may be found just in specific cell types or are distributed in variety of cells and tissues. For instance, Table 1 summarizes major calcium-binding proteins present in the nervous system [7].

Three major classes of Ca^2+^-sensing structural modules have been identified as EF-hands [11], C2 domains, and annexin folds [12].

The EF-hand domain is one of the common known motifs to bind calcium to proteins [13, 14]. Falke et al. [15] and Linse and Forsén [16] have shown the finely tuned metal-binding ability of the EF-hand motif. Sensitivity to minor changes in amino acid sequence enables this motif to exhibit a range of Ca^2+^ affinities functionally matched to the role of each EF-hand-containing protein. The affinity observed is affected by intramolecular interactions, since, owing to contacts with other EF-hand motifs, Ca^2+^ can bind in a cooperative manner as well as by the intermolecular interactions formed with target proteins. The EF-hand motif consists of two *α*-helices that are perpendicular to each other and a binding loop that actually provides the coordination oxygen atoms for the binding of Ca^2+^.

Although the highly conserved EF-hand motif has been studied extensively, non-EF-hand sites exhibit much more structural diversity which has inhibited efforts to determine the precise location of Ca^2+^-binding sites, especially for sites with few coordinating ligands.

A large number of C2 domain proteins are involved in Ca^2+^-dependent cell regulation role [11]. C2 domains (~130 residues) are also a structural module which function in a Ca^2+^-dependent membrane binding functionality and thereby serve as Ca^2+^ effectors for diverse Ca^2+^-mediated cellular processes [17]. Extensive studies of C2 domains have shown that, due to their structural diversity, C2 domains have disparate Ca^2+^ sensitivity. The Ca^2+^-binding sites of canonical Ca^2+^-dependent C2 domains are composed of three Ca^2+^-binding loops (CBL1–3) located at one side of the domain and both side chains (mostly Asp) and the peptide backbone are involved in coordination of multiple Ca^2+^ ions. Removal or introduction of key Ca^2+^-binding residues of C2 domains by mutation has been shown to convert Ca^2+^-dependent C2 domains to Ca^2+^-independent ones or vice versa. However, a recent study on rat and fly synaptotagmin-IV C2 domains showed that despite high sequence homology these C2 domain orthologs have distinctively different Ca^2+^-binding properties due to different orientations of critical Ca^2+^ ligands. This cautions the idea that purely sequence-based prediction of the Ca^2+^ affinity of C2 domains could be possible [17].

Considering the important role of these proteins in occurrence and/or diagnosis of many diseases a more detailed survey on the basic chemical specifications and principles of their calcium binding can provide biochemical insights that can lead to more fundamental understanding of how calcium interacts with protein motifs. In this study, we focus on investigation of the binding mechanisms and identification of crucial elements and active groups affecting calcium affinity of peptides which can provide more accurate methods for detection and quantification of calcium-binding peptides and proteins.

## 2. Experimental

Peptides were obtained all from PepScan Presto (Lelystad, The Netherlands) and Thermo Fisher Scientific GmbH (Ulm, Germany). Peptide stock solutions were prepared as 10^−4^ M in water (Milli Q, Milford, MA, USA) and the concentration of CaCl_2_ (Sigma Aldrich, USA) was 10^−3^ M in water. The ESI mass spectra (MS) and collision induced dissociation spectra (MS/MS, collision gas helium) were obtained in the positive and negative ion modes by direct infusion (4 *μ*L/min) of the peptide solution with or without CaCl_2_ using 10% (v/v) of isopropanol (BioSolve, Valkenswaard, The Netherlands). Potassium chloride was purchased from Sigma Aldrich, USA.

The spectra were obtained using an ESI-ion trap mass spectrometer (Bruker Daltonics, Bremen, Germany). A crucial parameter appeared to be the capillary current: this has to be kept below 30 nA, corresponding to a capillary voltage of approximately 4,000. If the current exceeds 30 nA, the metal complexes are destroyed [18], resulting in background signals only. The MS/MS spectra were recorded with the trap drive set optimally for the doubly charged precursor ion.* Ab initio* calculations were performed with the CBS-QB3 model chemistry [19] using the Gaussian 09 (Rev. B.01) suite of programs [20].

## 3. Results and Discussion

### 3.1. Experimental Approach

During a mass spectrometric quality assessment (by high resolution Matrix-Assisted Laser Desorption/Ionization-Fourier Transform-Ion Cyclotron Resonance-Mass Spectrometry (MALDI-FTICR-MS)) of synthetic 15-mer peptides of an onconeural protein HuD protein-spanning peptide pool, we noticed [21] that several peptides display an extraordinary large affinity towards Ca^2+^. This was evident from the intense peaks present at 37.9470 Da (corresponding to (P-H)^−^ *⋯*Ca^2+^) higher than that for the protonated peptide (PH^+^) as opposed to the usually found lesser intense peaks 37.9559 Da higher for the K^+^ adducts (corresponding to P*⋯*K^+^). The most intense Ca^2+^ adducts were found for the 15-mer peptides QSLGYGFVNYIDPKD (#22), TGATTDDSKTN (#9), GFVTMTNYDEAAMAI (#86), and MTNYDEAAMAIASLN (#87) of which the latter two are overlapping 15-mer peptides and show the most intense signals for the Ca^2+^ adducts. The common domains of peptides 86 and 87 contain the TNYDE sequence and so the 7-mer GTNYDEG and several other 7-mers (AGGGDEG, GGGGDEG, GTGGDEG, GGGGDEN, TTTTDEG, NGTYDEG, and QGTYDEG) were purchased in the above study as model compounds. In the present work we investigated by mass spectrometry the relative affinities of these peptides and additional peptides towards calcium. In this way we wished to ascertain the influence of different amino acid residues on calcium binding. To this end, we attempted to assess the relative affinities by employing a bracketing variant of Cooks' kinetic method [22, 23], by generating in an ESI source Ca^2+^ bound dimers of the type P_1_ *⋯*Ca^2+^ *⋯*P_2_. It is possible to apply the kinetic method in its simplest form, that is, a bracketing method, which makes use of only one or a few reference compounds with known thermodynamic properties and assumes no entropy effects in the dissociation reactions [24, 25].

**Figure 1:**
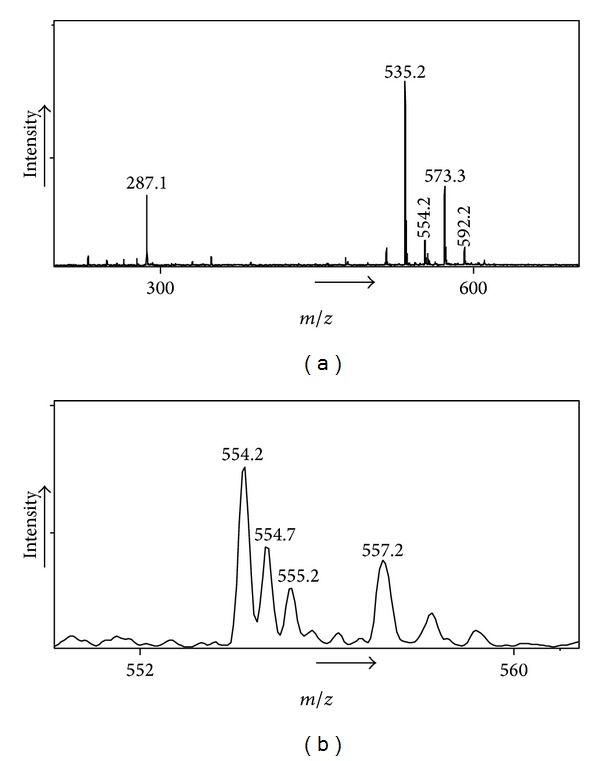
(a) ESI-ion trap (direct infusion) mass spectrum of GTGDEG in the presence of Ca^2+^. (b) Insert showing peaks for P*⋯*Ca^2+^ *⋯*P (doubly charged) at *m*/*z* 554.2 and for P*⋯*Na^+^ at *m*/*z* 557.2 (singly charged).

The ESI mass spectrum obtained by direct infusion of GTGDEG (further represented as P) in the presence of Ca^2+^ contains a clear peak for P*⋯*Ca^2+^ *⋯*P at *m*/*z* 554.2 with the isotope peaks separated at 0.5 Da as expected for doubly charged ions; see Figure 1. At 3 Da higher (irrespective of the mass of P), peaks are found for the singly charged adduct P*⋯*Na^+^. The signals for P*⋯*Ca^2+^ *⋯*P (and also that for P*⋯*Ca^2+^ (*m*/*z* 287)) disappear when the sample is replaced by a solution of GTGDEG with extraneous K^+^. This shows that the peak at *m*/*z* 554.2 is indeed P*⋯*Ca^2+^ *⋯*P and not the isobaric species ^+^PH*⋯*K^+^ *⋯*P. Next the species P*⋯*Ca^2+^ *⋯*P was mass selected and subjected to collision induced dissociations; it was found that the dissociation to P*⋯*Ca^2+^ + P was very weak and instead the precursor ion dissociates abundantly by proton transfer to two singly charged products, which in the MS/MS mass spectrum give rise to two signals for [(P-H^+^) + Ca^2+^] and for PH^+^ of near equal intensity, P*⋯*Ca^2+^ *⋯*P→[(P-H)^−^ *⋯*Ca^2+^] + PH^+^. (Alternatively P*⋯*Ca^2+^ *⋯*P may already be present as a species where one P is deprotonated and the other P is protonated). Heterodimers of the type P_1_ *⋯*Ca^2+^ *⋯*P_2_ can be similarly made from a mixture of the peptides P_1_ and P_2_ and CaCl_2_. Thermochemical arguments (see Supplementary Material Derivation of equations (1) and (3) available online at http://dx.doi.org/10.1155/2014/153712) along the lines of Nemirovskiy and Gross [26] show that the respective signal strengths of the product ions from the heterodimers P_1_ *⋯*Ca^2+^ *⋯*P_2_ (i.e., [(P_1_-H)^−^ *⋯*Ca^2+^]) and P_2_H^+^ compared to [(P_2_-H)^−^ *⋯*Ca^2+^] and P_1_H^+^ are governed by the quantity Δ_p_:

(1) $$\begin{array}{c} \Delta_{\text{p}}=\Delta {\text{Ca}}_{\text{a}\;\text{ff}}{\left(\text{P-H}\right)}^{-}-\Delta \text{PA}{\left(\text{P-H}\right)}^{-}-\Delta \text{PA}\left(\text{P}\right), \end{array}$$

where ΔCa_a ff_ is the difference in calcium affinity and ΔPA is the difference in proton affinities (see supplementary information). If the differences in PAs can be neglected, then a calcium affinity ladder for [P-H]^−^ can be constructed. We also investigated the negatively charged dimer ions [P-2H]^2−^⋯Ca^2+^⋯[P-2H]^2−^ which were observed to fragment to

(2) [(P-3H)3−⋯Ca2+]and  to  [P-H]−,i.e.,  [P-2H]2−⋯Ca2+⋯[P-2H]2− ⟶[(P  - 3H)3−⋯Ca2+]+[P-H]−

paralleling the observations for the positively charged dimer ions; in this case, the product ion intensities (see supplementary material) are governed by the quantity Δ_*n*_:

(3) $$\begin{array}{c} \Delta_{n}=\Delta {\text{Ca}}_{\text{a}\;\text{ff}}{\left(\text{P-}3\text{H}\right)}^{3-}-\Delta \text{PA}{\left(\text{P-}3\text{H}\right)}^{3-}-\Delta \text{PA}{\left(\text{P-}2\text{H}\right)}^{2-}. \end{array}$$

**Figure 2:**
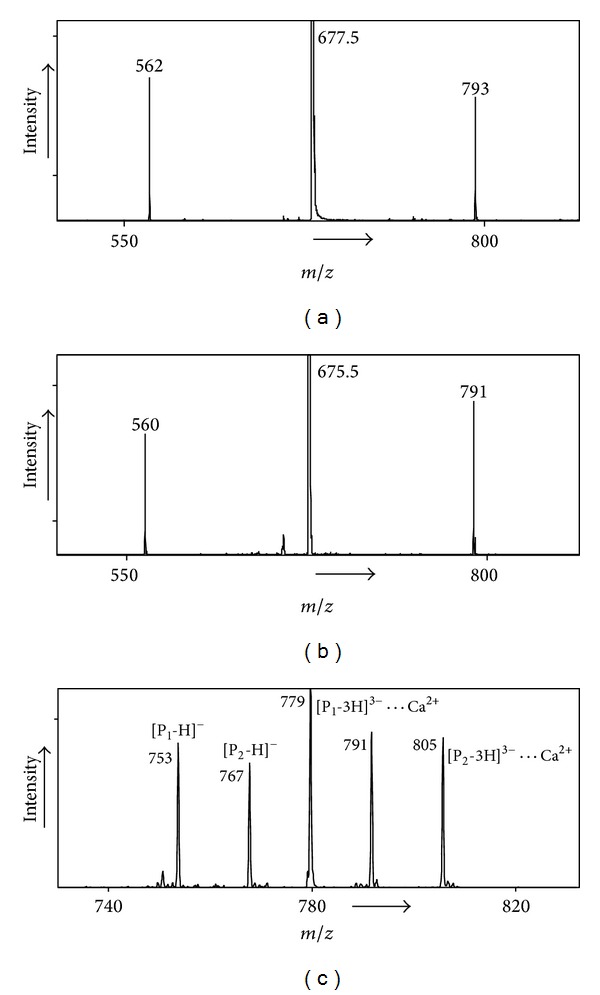
(a) MS/MS spectrum of *m*/*z* 677.5 precursor of the homodimer (P_1_ ⋯ Ca^2+^ ⋯ P_2_): P_1_ = GTYDEGN and P_2_ = AGGGDEG; (b) MS/MS spectrum of *m*/*z* 675.5 precursor of the homodimer (P_1_-2H)^2−^ ⋯ Ca^2+^ ⋯ (P_2_-2H)^2−^; (c) MS/MS spectrum of *m*/*z* 779.3 precursor (P_1_-2H)^2−^ ⋯ Ca^2+^ ⋯ (P_2_-2H)^2−^: P_1_ = NGTYDEG and P_2_ = QGTYDEG. No preference for Ca^2+^.

In Figures 2(a) and 2(b) an example is shown of such an experiment for a heterodimer, both in the positive and in the negative ion modes for P_1_ = GTYDEGN and P_2_ = AGGGDEG. It can be seen that in both cases the calcium ion prefers the peptide GTYDEGN as opposed to AGGGDEG. (For the higher energy process (not shown) leading to P⋯Ca^2+^ + P there, too, is a preference for GTYDEGN.) Thus for both (P-H)^−^ and (P-3H)^3−^, calcium prefers GTYDEGN over AGGGDEG. Note that all the above peptides contain three acidic functionalities (the primary binding sites for calcium) leading to a maximum negative charged state of three. Hence differences in calcium affinities of (P-3H)^3−^ reflect differences in calcium interaction with nonacidic residues.

Figure 2(c) shows an example of a heterodimer [P_1_-2H]^2−^⋯Ca^2+^⋯[P_2_-2H]^2−^, with P_1_ = NGTYDEG and P_2_ = QGTYDEG, where products are formed with the calcium ion equally probable on both (P-3H)^3−^ peptides. Thus, replacing the amino acid residue N by the higher homologue Q has no effect on the product ion distribution. For the negative ions we obtain by bracketing [22, 23] the following calcium affinity order for the 7-mers: AGGGDEG = GGGGDEG < GTGGDEG = GGGGDEN < TTTTDEG < NGTYDEG = QGTYDEG = GTYDEGN. Thus AGGGDEG and GGGGDEG have similar affinities which are less than those for GTGGDEG and GGGGDEN which in turn show less affinity in comparison to TTTTDEG. The peptides NGTYDEG, GTYDEGN, and QGTYDEG have the largest calcium ion affinities among the 7-mers. The above results lend great support for our previous proposal that the amino acids T and Y (also N and Q) strongly influence calcium binding as observed by the largest binding found for NGTYDEG, QGTYDEG, and GTYDEGN. (Since GTYDEGN and QGTYDEG are susceptible to deamination, as evidenced by an intense peak for loss of NH_3_, the peptide NGTYDEG was used in further studies; see below.) Note that both T and Y have hydroxyl functionalities which have been proposed to increase calcium binding [21].

### 3.2. Relative Affinity Assays

Before extending our studies to larger peptides, it should be noted that the above derived affinity order reflects the amino acid sequence in peptides having the same number of amino acids. When the peptides get longer, more potential calcium binding sites are possible even if no additional functional groups (in addition to the longer backbone) are present. A case in point is provided by the pair GTGDEG (a 6-mer) and GTGGDEG (a 7-mer). It was found that the dimer ion [P_1_-2H]^2−^ *⋯*Ca^2+^ *⋯*[P_2_-2H]^2−^ where P_1_ = GTGDEG and P_2_ = GTGGDEG fragments almost exclusively to [(P_2_-3H)^3−^ *⋯*Ca^2+^] + [P_1_-H]^−^ and so the larger peptide ion accommodates the calcium ion more efficiently. That is to say, a size effect exists and so interpretation of the following results should be performed keeping such size effects in mind.

Using NGTYDEG as a reference (P_1_) we investigated a series of 24 additional peptides (P_2_) from 7-mers to 19-mers, see Table 2, by monitoring the dissociations from the negatively charged heterodimer ions [P_1_-2H]^2−^ *⋯*Ca^2+^ *⋯*[P_2_-2H]^2−^. In the following, we briefly discuss the results in Table 2 and we will refer to the entry numbers mentioned in this table.

For the 7-mers containing the same number of acidic residues (two), the following is observed. Substitution of T in the model peptide (NGTYDEG) by a G (NGGYDEG) reduces the relative calcium affinity (entry 1) due to loss of the OH functionality, but with an S amino acid instead of T (NGSYDEG versus NGTYDEG) the affinity towards calcium remains the same (entry 2), which is expected due to preservation of the OH functionality. Substitution of N in the model peptide (NGTYDEG) by a G (GGTYDEG) also caused diminished affinity (entry 3). These results show the importance of the residues T, S, and N for efficient calcium binding. In general, the absence of such amino acids for coordination (such as T or S) revealed reduced affinity; see entries 4–8.

**Figure 3:**
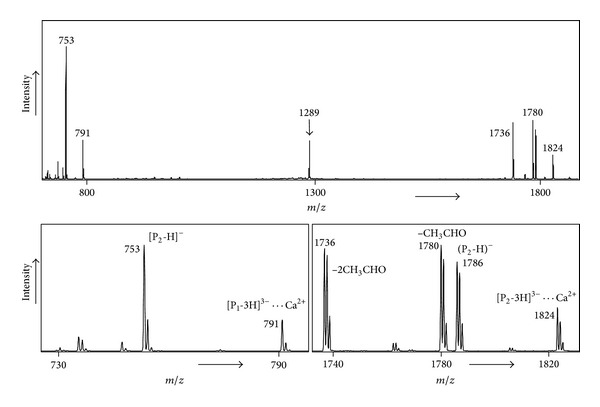
MS/MS spectrum of *m*/*z* 1289 precursor (P_1_-2H)^2−^ *⋯*Ca^2+^ *⋯*(P_2_-2H)^2−^: P_1_ = NGTYDEG and P_2_ = SLGHTLFGDKLGGGGTVAT.

**Figure 4:**
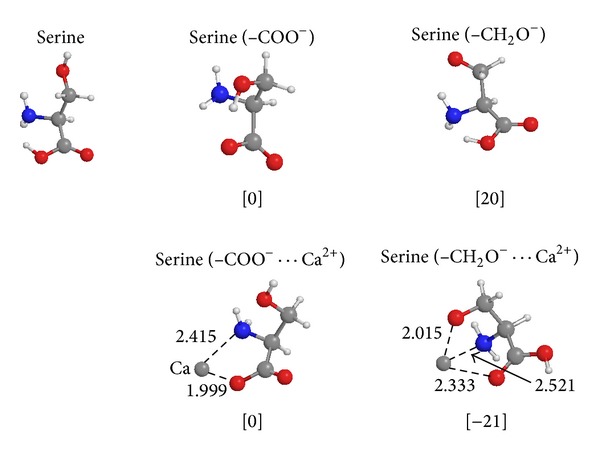
Calculated structures and energies (CBS-QB3) for serine, serine deprotonated at COOH and serine deprotonated at CH_2_OH in the absence and presence of Ca^2+^.

For these negatively charged heterodimer ions [P_1_-2H]^2−^ ⋯ Ca^2+^ ⋯ [P_2_-2H]^2−^ we observed no [(P_2_-3H)^3−^ ⋯ Ca^2+^] fragments if P_2_ contains no or only one acidic amino acid residue, as in the 7-mers listed in entries 9–12. This also holds for larger peptides (see entries 13–22) and even for very large peptides, for example, ARRHPYFYAPELLFFAK (entry 21). This is so because these peptides have only one or two carboxylic functionalities and thus these peptides cannot produce the (P-3H)^3−^ ions necessary for calcium binding in our mass spectrometry based experiments. Even a small peptide, such as our reference peptide NGTYDEG, can produce such ions by deprotonation of all three carboxylic functionalities. However, when multiple T and S residues are present as in SLGHTLFGDKLGGGGTVAT (entry 23) we observe in the MS/MS clearly fragmentation to [(P_2_-3H)^3−^ ⋯ Ca^2+^] in competition with formation of [(P_1_-3H)^3−^ ⋯ Ca^2+^]; see Figure 3. This indicates that at least one other nonacidic amino acid can undergo deprotonation. In addition, we observed intense losses of one and two CH_3_CHO molecules from [(P_2_-3H)^3−^ *⋯*Ca^2+^]; see Figure 3; these losses most likely occur from the threonine residue [21] which must have become deprotonated in order to shed CH_3_CHO [27]. This result indicates that two of the three threonine residues in peptide 23 can become deprotonated by Ca^2+^ in competition with deprotonation of a carboxylic functionality. In order to ascertain whether such calcium induced deprotonation of threonine (and of serine [21]) is feasible energetically we have performed* ab initio* calculations on the deprotonation of serine, as a model for larger peptides; the results of these calculations are summarized in Figure 4. We find that the gas-phase deprotonation energy (DPE) of the CH_2_OH functionality of serine is 20 kcal/mol higher than that for the COOH group (paralleling the general observation that DPEs of simple acids are lower than those of simple alcohols [28]); surprisingly, however, the reverse is true when calcium interacts with these functionalities; see Figure 4: the structure where Ca^2+^ interacts with the deprotonated –CH_2_O^−^ moiety lies 21 kcal/mol lower than the one where Ca^2+^ interacts with the –COO^−^ group, the opposite of the situation in the absence of Ca^2+^.

We conclude that such facile calcium induced deprotonation reactions from the nonacidic residues serine and threonine rationalize the observed large calcium affinity for peptide 23, although it contains only one acidic residue. We envisage that the Ca^2+^ ion attached to a carboxylic functionality can transport a proton from a serine or threonine residue to the peptide backbone chain. For example, according to our calculations, the gas-phase deprotonation energy of methanol, CH_3_OH, see (4), is 383 kcal/mol (compared to experimental, 382 kcal/mol [28]):

(4) $$\begin{array}{c} {\text{CH}}_{3}\text{OH}⟶{\text{CH}}_{3}{\text{O}}^{-}+{\text{H}}^{+} \end{array}$$

whereas that for the reaction

(5) CH3OH+Ca2+⋯OOCH− ⟶CH3O−⋯Ca2+⋯OOCH−+H+

is only 193 kcal/mol, which is well below the gas-phase basicities of peptides [29]; this would allow the proton to be transported from the serine or threonine residue to the peptide backbone. A bidentate structure as shown in (5) would lead to increased calcium binding.

Except for the model peptides NGTYDEG and GTYDEGN, the effect of ordering of the residues in other isomeric peptides was not studied in detail. Because such peptides have exactly the same masses, they cannot be distinguished by mass measurements. In such cases an intermediary peptide of different mass should be chosen (e.g., QGTYDEG) as reference for both peptides. Thus the relative affinities of NGTYDEG and GTYDEGN were determined through the intermediary of QGTYDEG; see above. Since many combinations are possible even for a selection of amino acids, this will require a substantial experimental effort and current experiments towards this end are in progress.

### 3.3. Selectivity Assays

To assure that this binding affinity of the peptides towards calcium is not a random coordination but a selective binding, we designed selectivity assays by preparing peptide mixtures and tracking the calcium bound peptide in the mixture in competition with other peptides present. The mixtures contain the peptides showing high and low relative calcium affinities; see above.  Table 3 summarizes the composition of the peptide mixtures. The binding preference of the calcium can be followed from MS/MS experiments.

As can be seen from Table 3, in mixtures 1 and 2, the peptide NGTYDEG remains the one having the strongest affinity towards calcium. In mixture 3 all the parties present containing the glutamic and aspartic acid residues are capable of strong calcium coordination.

### 3.4. Dimerization in Solution versus Dimerization in the Gas Phase

For strong calcium binding, it may be expected that the above dimers may also be present in solution and not only in the gas phase as observed with the mass spectrometer. To ascertain whether calcium binding also occurs in solution, we studied dimerization processes using NGTYDEG. To this end the negatively charged species [P-2H]^2−^ − Ca^2+^ − [P-2H]^2−^ was used as it produced intense signals in ESI. Direct infusion experiments were performed with 10 pmol/*μ*L of peptide and various parameters were investigated for optimum dimer signal strength, such as solvent composition (10% isopropanol), nature, and amount of salt (100 pmol/*μ*L CaCl_2_). Under these optimum conditions the effect of the pH was investigated; namely, pH = 3 (0.1% TFA), pH = 7 (water), and pH = 9 (0.1% TFA adjusted with acetic acid). It was found that signals for the dimers were observed only in nonacidic conditions with a larger intensity at pH = 7 and this indicates that the dimers are formed, at least partially, in solution prior to direct infusion. A hydrophilicity analysis reveals that, at pH = 7, NGTYDEG should be doubly deprotonated and this explains the relatively large abundance of the dimer in solution at pH = 7 and its absence at pH = 3 which is close to its isoelectric point at pH = 3.55.

## 4. Conclusions

Overall the investigated selective calcium binding of peptides can be characterized by a number of essential criteria. Glutamic and aspartic acid residues are responsible for the metal coordination in the first level of binding. In other words, the peptides are stabilized by Ca^2+^ binding to sites including anions associated with glutamate and aspartate. The OH functional group as in threonine (T) or serine (S) provides an extra coordination site via the oxygen of the OH group. In addition, transfer of the OH proton from T and/or S to the neighboring amino group enhances the coordination capacity of the peptide. The amino acids asparagine (N) and tyrosine (Y) can also favor the desired binding by increasing potential binding sites. This knowledge leads to a better understanding of the binding to and the detachment of calcium from peptides and proteins. In addition, our findings can be applied directly to the design of carrier materials to study calcium binding in peptides and proteins.

## Supplementary Material

Supplemental text: provides the derivation of equations (1) and (3) using the quantities Proton Affinity (PA) and Calcium ion affinity (Ca_aff_).

## Figures and Tables

**Table 1:** Major calcium-binding proteins in the nervous system.

Present in most cell types, including neurons	Present in certain cell types in CNS
**EF-hand family**	**EF-hand family**
Calmodulin [30] (ubiquitous calcium-dependent modulator of protein kinases and other enzymes)	Parvalbumin [31] (in some neurons)
Calpains [32] (calcium-dependent proteases)	Calbindin-D28K [33] (in some neurons)
*α*-Actinin [34]	Calretinin [33] (in some neurons)
**Other families**	Recoverin, visinin [35] (in photoreceptors; regulating guanylyl cyclase)
Annexins [36] (Ca^2+^-phospholipid-binding proteins of unknown function, but implicated in exocytosis)	S100*α* and S100*β* [9, 36] (in glia; effects on phosphorylation and neurite outgrowth)
Protein kinase C [37]	

**Table 2:** Extended list of the investigated peptides (7-mers to 19-mers) used in relative affinity experiments. The stronger partner is represented in upper case format and the weaker partner in lower case. Reference peptide partner = NGTYDEG.

Entry	Peptide
1	nggydeg
2	NGSYDEG ≈ NGTYDEG
3	ggtydeg
4	ggggdeg
5	ggggdea
6	agggdea
7	ggggden
8	agggdeg
9	aggyggg
10	agtyggg
11	gtygggn
12	gtyllgn
13	fqnallvr
14	slhtlfgdk
15	hpdysvvlllr
16	rhpdysvvlll
17	arrhpdysvvllr
18	rhpyfyapellffak
19	qhipsqhipsqhips
20	rhpdysvvlllrlakt
21	arrhpyfyapellffak
22	fqgnallvrytggggk
23	SLGHTLFGDKLGGGGTVAT ≈ NGTYDEG
24	GTYDEGQ

**Table 3:** Content of peptide mixtures. The calcium-bound peptides are presented as bold characters.

	Number 1	Number 2	Number 3	Number 4
Mix-1	**NGTYDEG**	FQNALLVR	AGGGEDG	
Mix-2	**NGTYDEG**	FQNALLVR	AGGGEDG	SLHTLFGDK
Mix-3	**NGTYDEG**	**GTYDEGQ**	**GGGGDEN**	
Mix-4	AGGGDEG	FQNALLVR	AEFAEVSK	
