# Supplementary material for: Dimerization of Peptides by Calcium Ions: Investigation of a Calcium-Binding Motif
Source: Int J Proteomics. 2014 Sep 14;2014:153712. doi: 10.1155/2014/153712 (PMC4177772; doi:10.1155/2014/153712)
Supplement: Supplementary file 1 — Supplemental text: provides the derivation of equations (1) and (3) using the quantities Proton Affinity (PA) and Calcium ion affinity (Caaff). [file 153712.f1.pdf]

Supplementary Information

Derivation of equations (1) and (2)

For the process: $P + H^+ \rightarrow PH^+$, the proton affinity (PA) is defined as: $PA(P) = \Delta H_f(P) + \Delta H_f(H^+) - \Delta H_f(PH^+)$

Similarly, for the process: $P + Ca^+ \rightarrow (P\bullet\bullet\bullet Ca^{2+})$, the calcium affinity ($Ca_{aff}$) is: $Ca_{aff} = \Delta H_f(P) + \Delta H_f(Ca^{2+}) - \Delta H_f(P\bullet\bullet\bullet Ca^{2+})$

1. $P_1\bullet\bullet\bullet Ca^{2+}\bullet\bullet\bullet P_2$ dissociating to $P_1 + (P_2\bullet\bullet\bullet Ca^{2+})$ or to $P_2 + (P_1\bullet\bullet\bullet Ca^{2+})$

$\Delta H_f(P_1\bullet\bullet\bullet Ca^{2+}\bullet\bullet\bullet P_2) \rightarrow \Delta H_f P_1 + \Delta H_f P_2 + \Delta H_f\, Ca^{2+} - Ca_{aff}\,(P_2)$

$\Delta H_f(P_1\bullet\bullet\bullet Ca^{2+}\bullet\bullet\bullet P_2) \rightarrow \Delta H_f P_2 + \Delta H_f P_1 + \Delta H_f Ca^{2+} - Ca_{aff}\,(P_1)$

Subtract:

$\mathbf{\Delta = \Delta Ca_{aff}\,(P)}$

2. $P_1\bullet\bullet\bullet Ca^{2+}\bullet\bullet\bullet P_2$ dissociating to $P_1H^+ + [(P_2\text{-}H)^- \bullet\bullet\bullet Ca^{2+}]$ or to $P_2H^+ + [(P_2\text{-}H)^- \bullet\bullet\bullet Ca^{2+}]$

$\Delta H_f\,[P_1H^+\bullet\bullet\bullet Ca^{2+}\bullet\bullet\bullet (P_2\text{-}H)^-] \rightarrow \Delta Hf(P_1H^+) + \Delta H_f[(P_2\text{-}H)^- \bullet\bullet\bullet Ca^{2+}]$

$\Delta H_f\,[P_1H^+\bullet\bullet\bullet Ca^{2+}\bullet\bullet\bullet (P_2\text{-}H)^-] \rightarrow \Delta Hf(P_2H^+) + \Delta H_f[(P_1\text{-}H)^- \bullet\bullet\bullet Ca^{2+}]$

or

$\Delta H_f\,[P_1H^+\bullet\bullet\bullet Ca^{2+}\bullet\bullet\bullet (P_2\text{-}H)^-] \rightarrow \Delta H_f P_1 + \Delta H_f H^+ - PA(P_1) + \Delta H_f\,(P_2\text{-}H)^- + \Delta H_f Ca^{2+} - Ca_{aff}[P_2\text{-}H]^-$

$\Delta H_f\,[P_2H^+\bullet\bullet\bullet Ca^{2+}\bullet\bullet\bullet (P_1\text{-}H)^-] \rightarrow \Delta H_f P_2 + \Delta H_f H^+ - PA\,(P_2) + \Delta H_f(P_1\text{-}H)^- + \Delta H_f Ca^{2+} - Ca_{aff}\,[P_1\text{-}H]^-$

Subtract:

$\Delta = \Delta H_f P_1 - \Delta H_f P_2 + \Delta H_f\,(P_2\text{-}H)^- - \Delta H_f\,(P_1\text{-}H)^- - PA\,(P_1) + PA(P_2) - Ca_{aff}\,[P_2\text{-}H]^- + Ca_{aff}[P_1\text{-}H]^-$

$\Delta = Ca_{aff}[P_1\text{-}H] - Ca_{aff}[P_2\text{-}H]^- - PA(P_1) - PA\,(P_2) + \mathbf{\Delta H_f P_1 - \Delta H_f P_2} + \Delta H_f\,[P_2\text{-}H]^- - \Delta H_f\,[P_1\text{-}H]^-$..........**(a)**

Also:

$\Delta H_f P_1 = \Delta H_f\,(P_1\text{-}H)^- + \Delta H_f H^+ - PA\,[P_1\text{-}H]^-$

$\Delta H_f P_2 = \Delta H_f\,(P_2\text{-}H)^- + \Delta H_f H^+ - PA\,(P_2\text{-}H)^-$

Subtract:

$\mathbf{\Delta H_f\, P_1 - \Delta H_f P_2} = -\,(PA\,(P_1\text{-}H)^- - PA\,(P_2\text{-}H)^-) + \Delta H_f\,(P_1\text{-}H)^- - \Delta H_f\,(P_2\text{-}H)^-$ **(b)**

Put (b) in (a):

$\Delta = Ca_{aff}\,(P_1\text{-}H)^- - Ca_{aff}\,(P_2\text{-}H)^- - (PA(P_1) - PA(P_2)) - (PA(P_1\text{-}H)^- - PA(P_2\text{-}H)^-) + \Delta H_f\,[P_2\text{-}H]^- - \Delta H_f\,[P_1\text{-}H]^- - \Delta H_f\,(P_1\text{-}H)^- + \Delta H_f\,(P_2\text{-}H)^-$

$$\Delta = \Delta Ca_{aff}\,[P\text{-}H]^{-} - \Delta PA(P) - \Delta PA\,(P\text{-}H)^{-} \qquad (1)$$

3. $(P_1\text{-}2H)^{2-}$ •••Ca^{2+} ••• $(P_2\text{-}2H)^{2-}$ dissociating to $[(P_1\text{-}3H)^{3-} + Ca^{2+}] + (P_2\text{-}H)^{-}$ or to $[(P_2\text{-}3H)^{3-} + Ca^{2+}] + (P_1\text{-}H)^{-}$

$\Delta H_f\,[(P_1\text{-}H)^{-}$ •••Ca^{2+} ••• $(P_2\text{-}3H)^{3-}] \rightarrow \Delta H_f(P_1\text{-}2H)^{2-} + \Delta H_f H^{+} - PA(P_1\text{-}2H)^{2-} + \Delta H_f(P_2\text{-}3H)^{3-} + \Delta H_f Ca^{2+} - Ca_{aff}\,(P_2\text{-}3H)^{3-}$

$\Delta H_f[(P_2\text{-}H)^{-}$ ••• Ca^{2+} ••• $(P_1\text{-}3H)^{3-}] \rightarrow \Delta H_f\,(P_2\text{-}2H)^{2-} + \Delta H_f\,H^{+} - PA(P_2\text{-}2H)^{2-} + \Delta H_f\,(P_1\text{-}3H)^{3-} + \Delta H_f\,Ca^{2+} - Ca_{aff}\,(P_1\text{-}3H)^{3-}$

Subtract

$\Delta = \Delta H_f\,(P_1\text{-}2H)^{2-} - \Delta H_f\,(P_2\text{-}2H)^{2-} - PA(P_1\text{-}2H)^{2-} + PA\,(P_2\text{-}2H)^{2-} + \Delta H_f\,(P_2\text{-}3H)^{3-} - \Delta H_f(P_1\text{-}3H)^{3-} - Ca_{aff}\,[P_2\text{-}3H]^{3-} + Ca_{aff}\,[P_1\text{-}3H]^{3-}$

$\Delta = Ca_{aff}[P_1\text{-}3H]^{3-} - Ca_{aff}[P_2\text{-}3H]^{3-} +$ **$\Delta H_f\,(P_1\text{-}2H)^{2-} - \Delta H_f\,(P_2\text{-}2H)^{2-}$** $+ \Delta H_f\,(P_2\text{-}3H)^{3-} - \Delta H_f\,(P_1\text{-}3H)^{3-}$ **(c)**

Also:

$\Delta H_f\,(P_1\text{-}2H)^{2-} = \Delta H_f\,(P_1\text{-}3H)^{3-} + \Delta H_f\,H^{+} - PA\,[P_1\text{-}3H]^{3-}$

$\Delta H_f(P_2\text{-}2H)^{2-} = \Delta H_f\,(P_2\text{-}3H)^{3-} + \Delta H_f\,H^{+} - PA(P_2\text{-}3H)^{3-}$

Subtract:

$\Delta H_f\,(P_1\text{-}2H)^{2-} - \Delta H_f\,(P_2\text{-}2H)^{2-}$ $= \Delta H_f\,(P_1\text{-}3H)^{3-} - \Delta H_f\,(P_2\text{-}3H)^{3-} - PA[P_1\text{-}3H]^{3-} + PA[P_2\text{-}3H]^{3-}$ **(d)**

Put (d) in (c):

$\Delta = Ca_{aff}[P_1\text{-}3H]^{3-} - Ca_{aff}[P_2\text{-}3H]^{3-} - (PA(P_1\text{-}2H)^{2-} - PA\,(P_2\text{-}2H)^{2-} + \Delta H_f\,(P_1\text{-}3H)^{3-} - \Delta H_f\,(P_2\text{-}3H)^{3-} - PA[P_1\text{-}3H]^{3-} + PA[P_2\text{-}3H]^{3-} + \Delta H_f\,(P_2\text{-}3H)^{3-} - \Delta H_f\,(P_1\text{-}3H)^{3-}$

$\Delta = Ca_{aff}[P_1\text{-}3H]^{3-} - Ca_{aff}[P_2\text{-}3H]^{3-} - (PA(P_1\text{-}2H)^{2-} - PA(P_2\text{-}2H)^{2-}) - (PA(P_1\text{-}3H)^{3-} - PA(P_2\text{-}3H)^{3-}$

$$\Delta = \Delta Ca_{aff}(P\text{-}3H)^{3-} - \Delta PA(P\text{-}2H)^{2-} - \Delta PA(P\text{-}3H)^{3-} \qquad (2)$$
